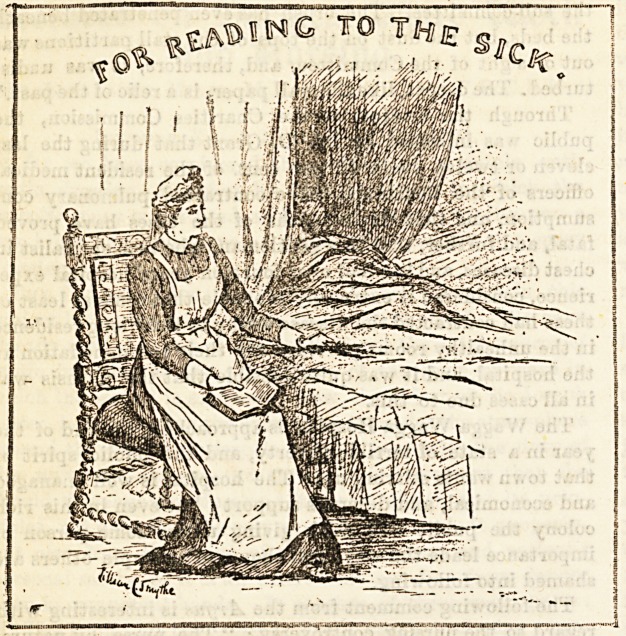# Extra Supplement—The Nursing Mirror

**Published:** 1891-01-17

**Authors:** 


Being the Extra Nursing Supplement of "The Hospital" Newspaper.

Contributions for this Supplement should be addressed to the Editor, The Hospital, 140, Strand, London, W.C., and should have the word "Nursing" plainly written in left-hand top corner of the envelope.

### En Passant.

ASHTON-UNDER-LYNE.—Miss A. M. Palmer, Matron of the Borough Hospital, Ashton - under - Lyne, wanted some books and toys to cheer her patients, but the guardians refused to supply these out of the rates, so Miss Palmer organised a bazaar which took place last week, and which brought in £60. The Mayor opened the bazaar, and the ladies of the town helped as stall-holders and also gave entertainments. Miss Palmer has worked at Hereford, St. George's, Oldham, and Preston and has everywhere been noted for her vigour and enthusiasm.

A PRIVILEGED PROFESSION. — A writer in the *Westminster Review* put forward the theory lately debated in the *Nightingale*, that at nursing schools as at other schools the pupils should pay to be taught, and be taken on in greater numbers. This is very nice on paper, but in practice the presence of a lot of girls learning in the wards would be most unpleasant. A knowledge of nursing can only be thoroughly gained by long and painful experience and by the running of considerable risk. In the past the theoretical side of nursing has been horribly neglected, but now the pendulum of public opinion is swung too far in the opposite direction, and we are threatened with neglect of practical training. Of the two the second evil is greater than the first. Nurses cannot be taught in "schools" the chief part of their craft; combine the lecture-room and the ward, grant nurses clinical instruction, and all will go well. At present a probationer can either pay or be paid; to make all probationers pay would be to place the profession out of the reach of those women who are most in need of work.

BELFAST NURSES' HOME.—An interesting account of the Belfast Nurses' Home and Training School appeared in the *Belfast Witness* for January 2nd. The writer stated that having been struck with the distinctive dark blue uniform seen in the streets, he was led to visit the home, which he describes as excessively cleanly. "In her year as a probationer the nurse is given board, lodging, washing, and uniform; for the next year she receives, in addition, £16; for the next, £17; and, if she should be engaged for a longer time, her wages rise at once to £22, increasing in time to £26. The Home at present numbers fourteen trained hospital nurses, twenty probationers, and thirty-four private nurses, which last do much important work attending to patients in the town, and anywhere else they may be summoned. There is a superannuation fund of about £36 a year in connection with the Home. An ideal nurse resembles an ideal soldier in many ways. Unquestioning obedience is her first duty; disregard of a superior's orders is quite as black a crime, and may lead to results quite as serious in kind as a soldier's disobedience to his officer. Again, the ideal nurse requires plenty of physical courage, nerve, and endurance; she must be as particular as any lancer or hussar in the care of her especial arms and accoutrements and the order of her quarters; she must observe military regularity and despatch in all her work; she must be always at hand when wanted, and always ready to throw herself into the breach in case of emergency, and, over and above all these soldierly virtues she must possess good temper, patience, gentleness, and self-forgetfulness in no small degree."

SHORT ITEMS.—Miss Morse, Matron of the Bath Hospital, Harrogate, was married on December 29th —Messrs. Gilbert and Hall, of Bournemouth has issued an "Invalid's Handbook" which they will send to any nurse on receipt of one stamp for postage. The book cantains some excellent recipes for invalid cookery.—Mrs. True, President of Sakurai-jo-gakko (girls' school), is planning to establish a sanatarium for the poor of Yotsuya, Tokyo, Japan. She also expects to build a Nurses' Training School adjoining it.—Our Philadelphia correspondent sends word that Dr. Weir Mitchell and Dr. W. W. Keen hope to start a Pension Fund for Nurses, similar to ours in the States.—The Countess of Zetland attended the Christmas fête at St. Joseph's Hospital, Dublin.—Six months ago a Clara Barton Training School for Nurses was started in conjunction with the Chicago Temperance Hospital; already the school has proved a success.

A QUEENSLAND SCHOOL.—We have before now referred with pleasure to the Nurse Training Schools of Victoria and New South Wales, and now, turning to another part of Australia, we find an excellent school at Brisbane, in connection with the hospital there. The hospital contains 227 beds and treats nearly 20,000 out-patients yearly. The Head Nurse is Miss E. F. Crosse, and under her are five charge nurses, ten staff nurses, 15 assistant nurses, and seven probationers. Candidates for the nursing staff can enter under two schemes, in one case they only sign for 18 months and receive £12 wages; in the second case they sign for three years and their pay begins at £20 and rises £5 every six months till it reaches £65. A certificate is given after 18 months if the probationer can satisfactorily pass an examination. The hospital is a handsome one and has lately been considerably enlarged by the erection of an out-patient department, theatre, &c. In the male wards there is a service of wardsmen who enter the service of the hospital at £40, rising £5 every six months to £70. The whole scheme for the nursing staff has been recently re-arranged.

GLASGOW GOES FORWARD.—At the New Year's meeting of the Glasgow Royal Infirmary nurses, a speech of great importance was made by Dr. William McEwen. Instead of looking back and making the old, old reference to "Sairey," Dr. McEwen looked forward, and saw in the future a faculty of nursing. Nursing was not yet in a perfect condition, and he asked whether it could not be raised to a distinct profession, with an entrance examination, with a minimum requirement, theoretical and practical, with its teachers and examiners, and its diplomas. Could not St. Mungo's College, with its omnivorous capacity, found a faculty of nursing? The material was at hand, the students were assembled, and were anxious and eager to begin. A nurse ought to have a good preliminary education, the more liberal the better. She ought to receive a fair knowledge of anatomy and physiology, bacteriology, the outlines of the practice of therapeutics, medicine, and surgery, sufficient to enable her to follow with intelligence the movements of the disease and the treatment she was entrusted to carry out. Dr. McEwen urged that every girl ought to be trained to be a useful member of society and to be self-supporting. She ought to be taught a profession, occupation, or trade, whereby she could earn her own livelihood, and, that done, she would be able to become a very much better member of society. He also alluded to the peculiar fitness of women for nursing, and pointed out how much man was indebted to woman. He remarked that a man required religion to make his life complete, and that requirement was intensified in woman. Nursing and religion should go together and become blended in her whole life, and then the nurse would be able to take a higher view of her duty and her profession. Dr. McEwen is one of the best friends nurses have in the United Kingdom, and this address of his, we are glad to hear, is to be published in full.

### Lectures on Surgical Ward Work and Nursing.

By Alexander Miles, M.B. (Edin.), C.M., F.R.C.S.E.

### Lecture X.—A SPRAY DRESSING.

So far we have considered the subject of a simple antiseptic dressing, meaning by that term one in which the carbolic spray is not used, and now I propose giving you a few hints and directions as to the management of a spray dressing.

The Carbolic Spray.—Perhaps I shall best begin this subject by saying a few words on the spray itself. We have already seen that one of the chief, if not the chief, source of septic infection is the atmosphere of the room or ward in which the wound is exposed. Now, the object of Sir Joseph Lister, in introducing the spray, was to render the atmosphere around the wound antiseptic. As it is usually the duty of the nurse to manage the spray, it is important that she should thoroughly understand its construction and its principles, not only that she may prepare and work it properly, but also from the point of view of her own and patient's personal safety, as it is an instrument not free from danger even in competent hands. It consists of three main parts: (1) The lamp; (2) the boiler; (3) the spray-producing apparatus. (1) *The lamp.*—This is a shallow, flat brass vessel arranged on the principle of an ordinary spirit lamp, with the wick projecting from the centre of the upper surface, and, in most cases, with a contrivance by which the size of the flame may be regulated. The lamp is filled with methylated spirit through an opening at the side, which is closed by a brass plug, or, better still, a screw. (2) *The boiler* is raised above the lamp on a light metal framework, the interspaces of which are filled in with fine wire gauze, to prevent the flame being blown into contact with any inflammable material. It consists of a metal tank, into which the water to produce the steam is put. Some sprays have a small glass window in the side through which the amount of water inside may be seen. On the top are (1) an opening through which the boiler is filled with water; and (2) a safety valve arrangement, which permits the escape of excessive steam when the spray is up. In some forms of spray these two parts are combined. You should be well acquainted with the mechanism of these parts, as it is by errors in the management of them that accidents so often happen. I need scarcely say that you should acquire your knowledge on the empty spray, as otherwise it may be dearly bought. (3) *The spray-producing apparatus.*—This consists of (one or) two fine nozzles which project from the upper part of the boiler, and which are provided with stopcocks. When these are turned on the steam rushes out through the narrow nozzle. By means of a needle projecting in front of these nozzles the steam is broken into a fine diffused spray. So far, however, this spray only consists of steam, which, although presumably aseptic, is certainly not antiseptic. The remaining part of the apparatus is that which supplies the carbolic acid. Connected with the under aspect of each nozzle is an india-rubber tube, which dips into a glass vessel attached to the boiler, and containing a 1 in 15 solution of carbolic acid. Now, when the steam rushes rapidly through the nozzle of the boiler, it creates a vacuum in the upper end of the elastic tubes, and, the carbolic rising to fill the vacuum, mingles in equal quantity with the steam, and is thrown out into the air in a fine antiseptic cloud of vapour. The strength of the carbolic in the air is thus 1 in 30, being diluted by the steam.

So much, then, for the engine itself. How is it worked? and what precautions are necessary to ensure safety to all concerned?

(I.) *The lamp.*—Always see that the lamp is full of spirit before you do anything else, and especially before you light it. I have seen a serious accident happen to a nurse, who, finding that the lamp required spirit, proceeded to fill it without extinguishing the flame. The result was that the whole of the spirit took fire, and the nurse was severely burned about the face and arms. Never let the first indication that the lamp is empty be its going out in the middle of a dressing. I have seen this occur repeatedly, much to the annoyance of everybody, especially of the nurse whose mistake it was. Always extinguish the light as soon as ever the dressing is over, otherwise the water in the boiler will boil out, the solder will melt, and the apparatus be ruined.

(II.) *The boiler.*—This should be filled about two-thirds full, and you should use water which is nearly boiling, as it saves time and spirit in getting up the spray. Never fill it quite to the top, as then there will be no place for the steam to collect, and the apparatus won't work. As I have just said in speaking of the lamp, you must never allow all the water to boil away, or the solder of the boiler will melt. Another important precaution you should take to ensure your own safety, while working the spray, is never to take out the plug at the top till the water inside has cooled down considerably. If you remove the plug while the boiler is full of steam, this will suddenly escape, and you may be severely scalded. I have known several nurses seriously injured by neglecting this precaution, and I cannot warn you too strongly with regard to it.

(III.) *The spray-producing apparatus.*—Of course you must see that the jar of carbolic (1 in 15) is kept full, and that the tubes are clear so that it can get freely into the nozzles. The tubes are very apt to get blocked by the particles of dust, wool, &c., sucked into them by the rush of fluid. This is simply obviated by tying a piece of fine gauze over the end of the tube. There are various ways by which you can tell that there is carbolic in the spray: (a) The *smell* of the vapour should indicate the presence of the antiseptic, but if carbolic is being used for other purposes you may be deceived. (b) The *taste* of carbolic is also characteristic. This you can detect by passing your finger through the spray, and then touching the tongue with it, or, better still, by simply passing your tongue through the cloud of vapour, when you will at once experience the not unpleasant sweetish taste of the acid if it be present. (c) The *colour* of the carbolic spray is whitish grey, compared to the electric blue of the plain steam. The contrast is exactly comparable to that of the smoke of a cigarette blown from the mouth, and that from the burning tobacco. (d) The hissing sound of the two sprays differs considerably, but this you can only learn by experience.

The last precaution I will give you here in connection with the working of the spray is never to turn the steam *directly* on to the patient. Always turn it on to your own sleeve first. While the spray is not working the nozzle gets filled with a quantity of very hot water, and when you suddenly turn the steam on to the patient you project this hot water with great rapidity on to his skin, causing him considerable pain. If you once have it projected on to your own face, there is little chance that you will ever turn it on to your patient.

### Death in Our Ranks.

On December 23rd, 1890, Eliza Ockenden, aged 40, at 37, Leader Street, Chelsea, from phthisis. Her mother and grandmother were nurses at St. George's, and although she was trained at Middlesex, she spent the last seven years of her life in faithful service to St. George's. The friends are pained because when the Matron was sent a notice of the death, the letter was not even acknowledged. Was it ever received?

### Notes from Australia.

*(By Our Own Correspondent.)*

Melbourne, November 14th.

THE Charities Commission has issued its first report of progress; so far it recommends:—

1. Removal of the depôts of the Immigrants' Home on the St. Kilda Road, Melbourne.

2. Establishment of a casual ward on the north bank of the Yarra, near Prince's Bridge.

3. Transfer of the permanent inmates now in the buildings on the St. Kilda road to the Royal Park.

4. Removal of the Benevolent Asylum to Cheltenham.

5. Removal of the Melbourne Hospital.

6. Erection of the Melbourne Hospital on the site known as the "Pig Market."

7. The immediate establishment of an Infectious Diseases Hospital.

8. The employment of female nurses in hospitals wherever practicable.

9. The establishment of a Board of Examiners, from whom nurses should obtain a certificate of competency.

10. The provision of better accommodation for nurses, relief from menial work, and the raising of their social status.

11. The passing of an Act to compel persons in good circumstances to support their indigent relations who are burdens on our charitable institutions.

12. Regular inspection of all the charitable institutions of the colony by competent firemen, and the enforcement of methods for the safety of the inmates of charitable institutions in case of fire.

13. The printing of all the reports of the inspector of charities as Parliamentary papers.

At least we have got through more work than your Lords' Committee.

The Intercolonial Charities Conference has just been held here. Professor Morris delivered the inaugural address, and mentioned General Booth's Scheme with appreciation, but thought his settlers would be a most undesirable immigration for Australia. Papers were read on Rescue Work, Ladies' Benevolent Societies, Charity Organization, Science and Charity, Pauperism—its Nature, Causes, and Remedies, and other subjects. Several of the papers were by women, and the fair sex also took its share in the debates. The next conference is to be held at Hobart, in 1891.

Our cry here just now is "Insanitary Melbourne!" and it is quite time it was raised. The number of deaths from typhoid and other fevers is undoubtedly due to the defective drainage, water supply, and want of fever hospitals. Dr. Gresswell has just presented a lengthy report (the result of three months' labours) on the subject to the Board of Public Health.

At the last meeting of the Committee of Melbourne Hospital, Mr. A. Webster gave notice of the following motion: "That in the opinion of this Committee it is desirable that the house now occupied by the Secretary be given to the Medical Superintendent, and that the rooms he occupies be placed at the disposal of the junior medical staff for sleeping accommodation." Mr. George Godfrey, referring to the inadequate sleeping accommodation provided for the medical staff, said that, unless steps were taken to improve it, action would be taken against the Committee by the health officer of the city. The Resident Medical Officers sleep two in a room; this is a description of one of the rooms: "It is gloomy and depressing in the extreme. Upon the chimney piece the boots of generations of medical officers have left their mark. The wall paper which delighted the gaze of the residents of 20 years ago remains as a legacy for the present ones. At any rate, there is no tradition as to its ever having been renewed. The ceiling is black with the smoke of thousands of pipes. The room has been swept and garnished preparatory to the visit of the sub-committee. The brush has even penetrated beneath the beds, but the dust on the tops of the stall partitions was out of sight of the Committee, and, therefore, it was undisturbed. The carpet, like the wall paper, is a relic of the past."

Through the medium of the Charities Commission, the public was informed by Dr. D. Grant that during the last eleven or twelve years 10 per cent. of the resident medical officers of the institution have contracted pulmonary consumption, and that five-sevenths of the cases have proved fatal, and further, that this gentleman, who is a specialist in chest diseases, and has had over fourteen years' hospital experience, considered it extremely probable that some at least of these had contracted the disease owing to their long residence in the unhealthy rooms provided for their accommodation at the hospital, and it was quite possible that the phthsis was in all cases due to this.

The Wagga Wagga Hospital is approaching the end of the year in a state of terrible poverty, and the public spirit of that town wants stirring up. The hospital is well managed and economical, and deserves support; but even in this rich colony the public will shirk giving unless some person of importance leads the way, and shows an example others are shamed into following.

The following comment from the *Argus* is interesting with regard to the nursing controversy: "The nurse, by natural instincts, by education, and by training should be a lady. We want no drowsy maids-of-all-work in the wards by night, and no toil-worn fingers torturing bared nerves in the operating-rooms by day. Tenderness, delicacy with all the firmness and endurance of accomplished womanhood are the qualities requisite for a nurse, and how shall these be bred and matured if all the surroundings of the nurse's life are as those of a kitchen or a scullery maid? It may be galling to our conceit, though it should be healthful to our earnest and honest resolve, to learn that "Old Sydney" is very far ahead of us in this way. While we are considering what ought to be done the *Builder* is able to publish an illustration of a nurses' home now being erected at the Prince Alfred Hospital, Sydney, and to publish a description of a handsome building to accommodate 55 sisters and nurses."

Hospital Sunday and Saturday have been kept here with considerable success and increased subscriptions. On Hospital Sunday a service was held in the Town Hall, conducted by the Rev. W. Stacey Chapman, the Bishop of Melbourne preaching. The collection was £63. Doth days were wet and gloomy, still the public supported the movement well, and the hospitals cannot complain.

### Appointments.

[It is requested that successful candidates will send a copy of their applications and testimonials, with date of election, to THE EDITOR, The Lodge, Porchester Square, W.]

BIRKENHEAD FEVER HOSPITAL.—We rejoice to hear that Miss Sidné Reed, of the Dumfries Royal Infirmary, has been appointed Superintendent of this hospital. There will be no more scandals now.

GRANTOWN.—Miss Mary Ellen Dix has been appointed Matron of Lady Seafield's private hospital at Grantown on Spey. Miss Dix trained at Brownlow Hill for three years, and secured her certificate of efficiency. Miss Dix has lately been working as Matron at the C.C. Poorhouse Hospital at Irvine, where she has given great satisfaction.

GORLESTON COTTAGE HOSPITAL.—Miss H. Lucy Gooden-Chisholm (late of the Boston Hospital, Lincolnshire) has been elected Matron of this institution, in the place of Miss C. B. Wilkie, resigned.

### ENCOURAGEMENT.

To encourage is to give confidence, to make a man bold to do what he naturally fears. A few people require very little or no encouragement; they are enough for themselves, while others again have such a very excellent opinion of their own merits that they rashly attempt things beyond their powers and "rush in where angels fear to tread." Of these we can only hope that in spiritual things they will see their error before it be too late. It is to the meek and lowly of heart, the humble and contrite of spirit to whom we are now alluding, and to whom Christ gives an express blessing and encouragement. He tells them that theirs is the Kingdom of Heaven. Not only in the next life but in this they enjoy that Kingdom which cometh not with observation. No man knows the peace and happiness which reign in their hearts, or the continual feast of the contented mind. They care not if their neighbour seems prosperous while they work hard for their living, and are not envious when he appears to be getting all the plums and leaving them nothing but the stones.

They are not always looking out for slights, and fancying people are insulting them; they never struggle for the uppermost place at a feast, nor the chief seat in the synagogue; they know that their heavenly Father has better things prepared for them that love Him, and they do love Him, and pray constantly that they may be able to show it by their lips and in their lives.

But these happy souls at times require a little encouragement. They have to grow in righteousness, and learn day by day to walk in God's ways. He sends them trials to strengthen their faith. In the Scriptures they will find many examples which they can ponder over with advantage.

Some people want to get rid of the toils of sin; they are willing, nay, anxious, to start in the narrow way which leads to eternal life. Let us encourage them like King Josiah to serve the Lord, for they are like the little child trying to make its first step to reach its mother. Who has not seen it put forth its tiny foot, then draw it back again in alarm. It tries once more, but dares not go alone; its heart fails, and it fears to make the effort. At length the smiling, loving mother stretches out her finger, the little one grasps it, rippling over with delight, and finds that easy which before seemed impossible.

The timid Christian can learn a lesson from the babe. You long to walk towards God, but shrink from the first step. You feel you cannot do it alone; that is out of the question; but encourage yourself in the Lord; He will strengthen the feeble knees and make the rough places plain. Grasp His loving hand, hold it fast, never let it go, and He will lead you safely through this world to the mansions of the blest.

### Everybody's Opinion.

[*Correspondence on all subjects is invited, but we cannot in any way be responsible for the opinions expressed by our correspondents. No communications can be entertained if the name and address of the correspondent is not given, or unless one side of the paper only be written on.*]

### CHRISTMAS FARE IN WORKHOUSES.

Miss Twining writes:—I regret to see the expression of opinion in a periodical which goes to many workhouse infirmaries, that it is unkindness or a "fad" to deprive the inmates of beer or porter on Christmas Day. As one who has had experience of both giving and withholding it, will you allow me to state the results of both? In the list published in the papers of 25 metropolitan unions and parishes, I find an increase in what I venture to think the right direction; six did not give the drinks named, while four offered alternatives, which, in the face of strong temptation, are hardly likely to be accepted. Others do not state the fact either way, so of these I cannot speak. For the last two years at Kensington, I am glad to say, a majority decided not to give it, and the testimony of our experienced master of twenty years was that never had so peaceful and comfortable a Christmas Day been passed. This was also stated by the master at Lambeth, and I may say of all others who have tried the plan. A nurse from a country union told me that all the bickerings and quarrellings of the day were caused by the beer, supposed to be a treat and a luxury, but the unaccustomed excitement of which naturally affected the weak bodies and minds of those who partook of what had probably, to many, been their ruin, and led to their becoming paupers. Other officers also at Kensington expressed their satisfaction at the change, for outsiders knew little of the mischief and bartering and deceit that resulted from the so-called treat. Surely this is the plain, common-sense view of the matter, when abundance of harmless food and extras are always allowed. The inmates are children, weak and unable to guide themselves, and they must be treated as such. May I venture to suggest that the "fad" and the sentimental side, is to suppose that there is no treat or pleasure for Englishmen and women without a "pint of beer?"

### ASYLUM ATTENDANTS.

"A. I." writes:—My opinion upon the notice set forth at Durham County Asylum, and signed "Robert Smith, M.D.," is that it is a very narrow-minded one.

I want to ask Dr. Smith whether all his experience has been gained at one institution, and if he is not desirous of a change? He wouldn't always like to think he was to remain in the same office, and never rise to a higher position.

As a mental nurse of twelve years' experience, I should certainly advise my fellow nurses to push ahead. It is well all medical superintendents do not set forth such "notices," for we know there are many of our fellow workers holding good appointments through the kindly help of their superior officers. There is not a calling so unrecognized as asylum attendants, and yet it's the most trying calling there is. No need to lay extra restrictions on those who follow it.

### LADIES' COMMITTEES.

"M. W. W." writes: A few weeks ago I read in The Hospital that an establishment for gentlewomen had to be closed, because a Ladies' Committee had dismissed the Matron without giving any reason for so doing. This is by no means a solitary case. One hears of such unjust treatment only too frequently, the reason sometimes being that one of the ladies has a friend whom she wishes to take the post. The reigning Matron must be got rid of. How? Probably by the agency of a disaffected nurse or probationer, or by some scheme, or ingeniously-laid plot. The other ladies are easily beguiled into believing that it is necessary for the well-being of the institution that the Matron should be dismissed, or, in more polite terms, "asked to resign." This is no imagination as many readers of The Hospital know to their cost. I know of three such instances quite recently. But I am thankful to say I have never heard of a Matron being treated thus by a gentlemen's committee.

### Keeping Christmas.

On Tuesday, December 30th, Christmas was kept at **Congleton Cottage Hospital.** Patients, late patients, and a number of poor children, numbering upwards of 200 were entertained to tea, nearly every room in the commodious building having its aspect changed from that of a sick room to a place of brightness, conviviality, and harmony. All this was done through the kindness and liberality of a number of ladies and gentlemen in Congleton and district. The Mayoress, with other influential ladies, presided at the tables. After tea several songs were given by ladies and gentlemen. Various games were indulged in, and together with a capital magic lantern entertainment, the evening was made a very pleasant one to all concerned. Father Christmas was impersonated by Mr. C. R. Hall, who brought in his bag numerous and useful articles of clothing for the patients, and an inkstand for the Matron, Miss Wagnall, as a present from the patients.

New Year's evening was spent very pleasantly by the patients at the **City Asylum, Birmingham,** when a Christmas tree was provided for them, the Head Nurse, Miss Mountford, and the other nurses, having collected sufficient money to give everyone two or three presents each.

The **Great Northern Central Hospital** had a large and varied entertainment for the patients on January 6th. Useful gifts of clothing had been sent by the Baroness Burdett-Coutts, the Duchess of Albany, and others.

At **Horton Infirmary** there was a musical evening, but owing to the inclement weather the visitors were few. A Christmas tree laden with useful gifts was provided, thanks to the efforts of the Matron, seconded by the assistance of the ladies of the town.

**Central London Sick Asylum, Fitzroy Square, W.**—On Thursday evening last a most successful entertainment was given to the patients in this institution by the Chaplain, the Rev. C. P. Baxter. There was a distinguished company of artistes present, who were much applauded, amongst whom may be mentioned Madame de Naucaze, the Misses Nellie Levey, Maude Chichester, Ida Agabeg, Houliston, Leslie Walker, and Eleanore Leyshon, who acted admirably the part of Helen, in the Helen and Modus scene from the "Hunchback" (Mr. Arthur Wellesley taking the part of Modus with much credit); Messrs. Percy Pinkerton, S. J. Thompson, W. J. Wells, &c. The instrumental music was rendered very efficiently by an orchestra of gentlemen conducted by Messrs. R. W. Tayler and H. D. Lodge. The ward was tastely decorated and illuminated with fairy and other lamps. The evening was a most brilliant and enjoyable one. Mr. Charles Hobbs, the Chairman of the Board of Managers, with his usual good nature, presided, and in an eloquent speech expressed the pleasure it gave him to be present at these annual gatherings, and to see how thoroughly they all enjoyed the entertainment provided for them.

At the **Louth Hospital** Miss Bradwell and her nurses achieved a very successful day. Presents were distributed in the morning, in the afternoon there was music, and in the evening guests and games. The people of Louth did not forget to supply the sick with dainty fare for Christmas Day.

Dr. Thomas, the Superintendent, presided at the annual concert and Christmas tree at the **Glasgow Royal Infirmary.** Many nurses and residents gave songs and recitations; in fact, everything was done with the kind intention of cheering the poor sufferers during their temporary sojourn in the Infirmary. The Matron, Miss Wood, had evidently taken the greatest trouble in arranging presents and decorating the Christmas tree which was stripped after the concert, most of the patients being cheered with a small gift, the children and women being all provided for. The evening was a great success, and after hearty cheers for Miss Wood, "Auld Lang Syne" was sung, the patients then returning to their wards. Let us hope that this will, in days to come, be a bright remembrance to many a sufferer and enable them to think kindly of their Christmas spent in the Royal Infirmary.

On the 8th inst. a gathering of an interesting nature took place at the **Cottage Hospital, Beverley.** About forty of those who had been treated at the hospital during the past year assembled on the invitation of the Matron, and tea was provided for them in one of the wards, the Matron being assisted by several of her friends. Songs and recitations and a magic lantern display most enjoyably whiled away the time after tea, and at the close e_ch of the guests was presented with a small article from a Christmas tree which had been placed in the ward.

Now a peep at our cousins across the water: At **Chicago County Hospital** over 500 patients and nurses were regaled with Christmas fare. They was an enormous tree, a maypole dance, and a concert.

At the **Mercy Hospital, Chicago,** Christmas opened with high Mass in the Sisters' chapel, at 6.30 o'clock. Father Stever, a Franciscan, was the celebrant. The chapel altars were decorated with white roses, and at the entrances to the sacristy were placed palms and banked cut flowers. Fifty-two patients and Sisters received the Holy Communion.

### Christmas Parcels.

Our readers may care to hear that at the meeting of the House Committee of the Westminster Hospital, on December 23rd, it was unanimously resolved that the best thanks of the Committee were due to the makers of the garments forwarded by the editor of The Hospital. The secretary, in sending this resolution, concluded thus: "Miss Pyne also desires me to send her special thanks." Miss Gray, of the South London District Nursing Association, wrote: "Miss Mansel has sent me a parcel of bed jackets and nightingales, made by the readers of The Hospital. They are most useful and acceptable Christmas gifts for our poor patients. I had just been wishing for such a gift for them. Please accept my most grateful thanks."

### Presentations.

The nurses of the Manchester Private Nursing Institution, Grosvenor Street, All Saints', Manchester, have recently presented their lady superintendent, Mrs. Nicholas, with a very handsome silk eider-down coverlet as a token of their love and esteem. This is the third gift Mrs. Nicholas has received from her nurses.

On Wednesday 7th, inst., Mr. E. Claude Kingsford, L.R.C.P. London, M.R.C.S., Senior House Surgeon Bolton Infirmary, was presented by the resident officers and nursing staff with a handsome gold watch suitably inscribed, as a mark of their esteem, and regret at his resignation after three years, and of gratitude for his kindness.

Upon his retirement from the office of Senior Resident Medical Officer at the Royal Free Hospital to take up the post of Surgical Registrar to St. Thomas's Hospital, Mr. E. C. Stabb was presented by the nurses with a handsome silver cigar case, engraved with his initials, as a token of their esteem and good will.

Miss K. Phillippa Hicks has been presented with a lamp by the resident staff of the Hospital for Sick Children, Gt. Ormond Street; the servants of that institution have also presented Miss Hicks with a clock.

The Lady Superintendant of the Northamptonshire Nursing Institution was much pleased and gratified on New Year's Day with the presentation of a handsome timepiece by her nurses as a token of their regard.

Glasgow Royal Infirmary.—Miss Bowman, the senior Night Superintendent, having resigned, she has been presented with a handsome travelling clock and ivory purse, with initials, as a token of the respect in which she was held by night nurses, past and present, and as a slight recognition of the help rendered by her to the nurses in their various duties.

### The Nurses' Bookshelf.

### A GUIDE TO MASSAGE.*

Nurses will find this a convenient guide to the principles and practice of massage. It is a small book, simply written; it makes no attempt at rivalling the lengthy treatises on massage, but is addressed solely to the nurse who is learning, or has just learnt the art. It is the sort of book it is well to keep by one to refresh the memory with at intervals, or to turn to when an unusual case of massage of the eye, or other difficult parts, is wanted. There are some excellent outline illustrations. The nurse who has devoted herself solely to massage, in the full scientific meaning of the term, would probably scorn this little book, but the trained nurse who has merely acquired a knowledge of modern massage will welcome an elementary and handy guide-book.

### THE CARE OF THE SICK.†

This book has attained the position of a standard work in Germany, and is sure to meet with a welcome in England. It is a handsome and handy volume, and the illustrations are excellent. Dr. Billroth has not written solely for nurses; he addresses himself to a wider public, namely, to all women on whom the care of the sick is likely to fall. This is where he strikes new ground, for he is not afraid of teaching housewives and mothers too much; he frankly goes through all the details of typhoid or diphtheria nursing, and ends with a really excellent chapter on physiology which in a small space comprises just so much knowledge of the structure and functions of the human body as every woman should know. There is also an excellent chapter on the observation and care of the mentally diseased, and considering how scarce is the literature addressed to attendants, we regard this as one of the best features of the book. In fact, Dr. Billroth touches on every necessary point, and dwells in detail on those of importance. It is seldom that we find a book like this, which contains neither too much nor too little. The translation is literal with a vengeance; we have not had the pleasure of seeing Dr. Billroth's book in the original, but evidently it is "unterlage" which has been translated "underlay," and "moorerde" which has been translated "moor-earth." With all due deference, we would state that neither "underlay" nor "moor-earth" are English words at all, and since this is likely to become a standard work in England, we hope in the next edition terms which are in common use amongst nurses will be used. For instance: Instead of moor-earth, peat; instead of underlay, draw-sheet; instead of fungus-spore, germ; instead of decubitus, bed-sore. On page 137 the terms narcotic and anæsthetic have been muddled; on page 128 hot-water poultice should be fomentation; the term bandaging is constantly used instead of dressing; bouillon should be beef-tea; cicatrices should be heals; and dry is constantly used instead of aired. On page 76 we are told "how to change under the sheet without requiring much movement of the patient." Of course, the meaning of Dr. Billroth was the changing of the under-sheet. We defy any doctor or nurse, man or woman, to make sense of the following sentence: "To place an underlay over the pillow, with pillows between it and the head of the bed, and at the foot-end (under the knees)." A large proportion of English doctors and nurses have served in German hospitals, and before the next edition of "The Care of the Sick" comes out we advise the translator to secure the services of such a one to explain nursing terms; this will lead to far better results than the study of Mayne's "Medical Vocabulary." The excessive use of italics throughout the book is very irritating and unnecessary. In conclusion, we give the headings of the various chapters to show on what an excellent scheme Dr. Billroth has worked, and how completely he covers the whole subject from the popular standpoint: The Sick Room; General Rules for the Care of Patients Confined to their Beds; On the Fulfilment of Medical Orders; Preparations for Operations and Bandagings; Observations and Care of Fever Patients; Nursing in Epidemics, Precautions against Infection, Disinfection; Care of Nervous Patients and Patients Mentally Diseased; Aid in Accidents; Food and Diet; the Structure and Functions of the Human Body.

* "The Nurses' Guide to Massage," by Samuel Hyde, L.R.C.P. (John Heywood, price 1s. 6d.)

† "The Care of the Sick, at Home and in the Hospital," by Dr. Th. Billroth. Translated by J. B. Endean. (Sampson Low, price 6s.).

### Notes and Queries.

To Correspondents.—1. Questions may be written on post-cards. 2. Advertisements in disguise are inadmissible. 3. In answering a query please quote the number. 4. If a private answer is desired, a stamped addressed envelope must be forwarded.

Notice.—We must really ask our correspondents to be more particular in enclosing name and address. Constantly we receive queries or letters with no name attached.

### Queries.

(27) *Attendants.*—Where should I apply to hear of vacancies in institutions for the insane, for instance, at Bethlem?—*Nurse.*

(28) *Home Wanted.*—For a paralysed man, aged 79. Could pay a small sum weekly.—*H. Y.*

### Answers.

*Brisbane and Others.*—Next week; our pages are so full just now.

*Esther Bayley.*—Beatrice Evershed, who trained with you, and who is now at Longview Ranche, Henly, Hays County, Texas, U.S.A., would like to hear from you.

*Adam and a Reader.*—You have forgotten to send your names.

*District Nurse's Bag.*—We beg to inform M. H. K. that a district nurse's bag is made by Messrs. Down Brothers, St. Thomas's Street, Borough, S.E., and by Messrs. Southall Brothers and Barclay, Birmingham. We advise M. H. K. to write to both.

(26) *A. G. Farrer.*—Two letters have been sent you by post.

### Amusements and Relaxation.

### SPECIAL NOTICE TO CORRESPONDENTS.

First quarterly word competition commenced January 3rd, 1891; ends March 28th, 1891.

Competitors can enter for *all* quarterly competitions, but no competitor can take more than *one first prize* or *two* prizes of any kind during the year.

Three prizes of 15s., 10s., 5s., will be given for the largest number of words derived from the words set for dissection.

Proper names, abbreviations, foreign words, words of less than four letters, and repetitions are barred; plurals, and past and present participles of verbs, are allowed. Nuttall's *Standard* dictionary only to be used.

N.B.—Word dissections must be sent in WEEKLY not later than the first post on Thursday to the *Prize Editor*, 140, Strand, W.C., arranged alphabetically, with correct total affixed.

The word for dissection for this, the THIRD week of the quarter, being

"SNOWSTORM."

| Names. | Jan. 8th. | Totals. | Names. | Jan. 8th. | Totals. |
|---|---|---|---|---|---|
| Reynard | 30 | 30 | H. A. S. | 26 | 26 |
| Reldas | 30 | 30 | Sister Jack | 26 | 26 |
| Tinie | 30 | 30 | Crystal | 26 | 26 |
| Patience | 30 | 30 | Woodbine | 25 | 25 |
| Jenny Wren | 30 | 30 | Madame B. | 25 | 25 |
| Agamemnon | 30 | 30 | Shakespeare | 24 | 24 |
| Wyamaris | 29 | 29 | Smyrna | 23 | 23 |
| E. C. | 29 | 29 | Southwood | 21 | 21 |
| Ecila | 29 | 29 | Gipsy Queen | 21 | 21 |
| Hope | 29 | 29 | Snowball | 19 | 19 |
| M. W. | 29 | 29 | Rita | 16 | 16 |
| Qu'appelle | 28 | 28 | Mortal | 16 | 16 |
| Nil Desperandum | 28 | 28 | Nurse Annie | 15 | 15 |
| Lady Betty | 28 | 28 | Carmen | 11 | 11 |

### Notice to Correspondents.

N.B.—Each paper must be signed by the author with his or her real name and address. A *nom de plume* may be added if the writer does not desire to be referred to by us by his real name. In the case of all prize-winners, however, the real name and address will be published.

## Figures and Tables

**Figure f1:**